# A Unique Presentation of the Glenoid, Coracoid, and Proximal Humerus Fractures

**DOI:** 10.7759/cureus.40358

**Published:** 2023-06-13

**Authors:** Angelos Assiotis, Harpal S Uppal, Adam Rumian, Clarence Yeoh

**Affiliations:** 1 Trauma and Orthopaedics, Lister Hospital, Stevenage, GBR

**Keywords:** upper extremity trauma, upper limb surgery, proximal humerus fracture, glenoid, coracoid process fracture

## Abstract

Fractures of the proximal humerus are common injuries with a bimodal age distribution. They usually present in younger patients after high-energy trauma and in elderly patients after lower-energy trauma. Fractures of the proximal humerus are rarely associated with concomitant fractures of the glenoid, and this is a complex injury pattern that indicates the presence of significant instability. Such injuries are usually treated surgically. Even more rarely, patients may present with proximal humerus fractures and fractures of the coracoid process. A male patient presented to our emergency department (ED) after a fall off the loading platform of his heavy goods vehicle (HGV), resulting in a right shoulder injury. During his initial assessment in ED, a computerised tomography (CT) scan demonstrated the presence of a comminuted proximal humerus fracture, a comminuted anterior glenoid wall fracture, and a coracoid process displaced fracture. Surgical fixation of all three fractures was undertaken in the same sitting. This is the first case described in the literature with a combination of the above injuries and serves as a reminder that as trauma complexity and incidence continue to increase, we should maintain a high index of diagnostic suspicion when dealing with such patients. Furthermore, we present our treatment approach for this case and the rationale behind it.

## Introduction

Proximal humerus fractures and glenoid concomitant fractures are rare injuries that are usually caused by high-energy trauma and present with significant shoulder instability [1]. Another rare traumatic presentation of the shoulder girdle is that of a proximal humerus greater tuberosity and coracoid process fracture [2], and this has been described as happening in the context of an anterior shoulder dislocation. A fracture of the coracoid in this context confers further instability, as it carries the origin of the conjoint tendon and also several ligamentous structures, such as the coracohumeral ligament and the coracoacromial ligament. In the case that we describe here, the challenge presented was one of significant shoulder girdle instability, as the patient had lost the buttress of the anterior glenoid wall and the stability conferred by the coracoid process and its structures. In addition to this, he had a highly displaced and comminuted varus fracture of the proximal humerus, rather than just a fracture of the greater tuberosity. When dealing with such complex cases, it is very important to diagnose such injuries on presentation so that appropriate surgical planning may take place.

## Case presentation

**Figure 1:**
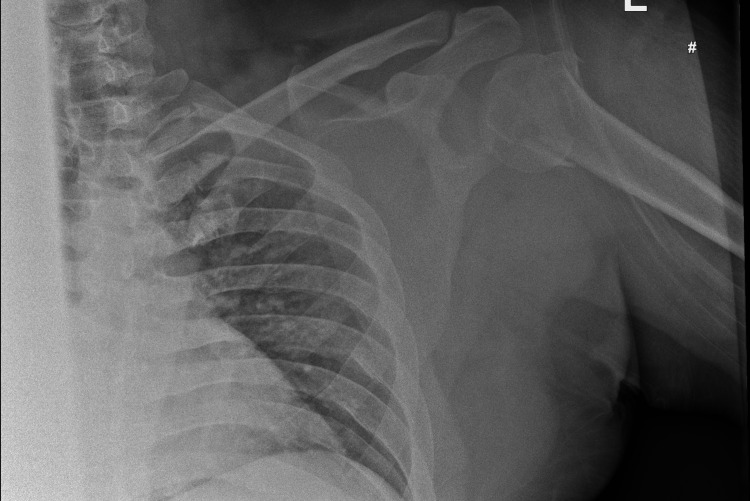
Anteroposterior radiograph of the left shoulder on presentation This demonstrates a varus-displaced comminuted proximal humerus fracture, but it does not clearly show the concomitant coracoid process and glenoid fractures.

A 37-year-old male patient without any comorbidities who works as a manual labourer presented to our ED after a fall off the loading platform of a heavy goods vehicle, landing heavily onto his left shoulder. He described that his shoulder felt like it was "out of place" as soon as he fell. He was in significant discomfort, and on assessment, he was found to have normal neurovascular function of the left upper limb. Initial radiographs demonstrated a highly comminuted, varus-displaced fracture of the proximal humerus (Figure 1).

**Figure 2:**
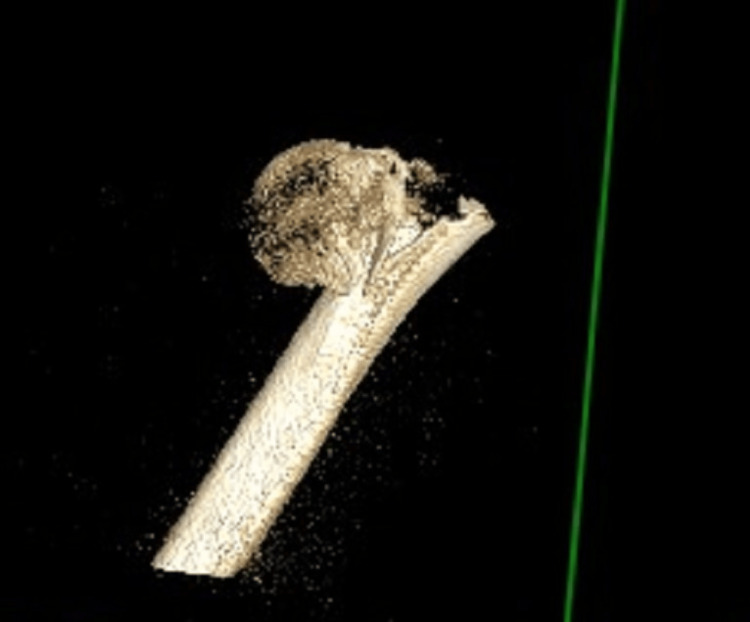
CT reconstruction of the proximal humerus fracture This demonstrates a highly comminuted and varus-displaced proximal humeral fracture. The lateral shaft fragment had lacerated the deltoid and had button-holed through it, as was noted in the operating theatre.

**Figure 3:**
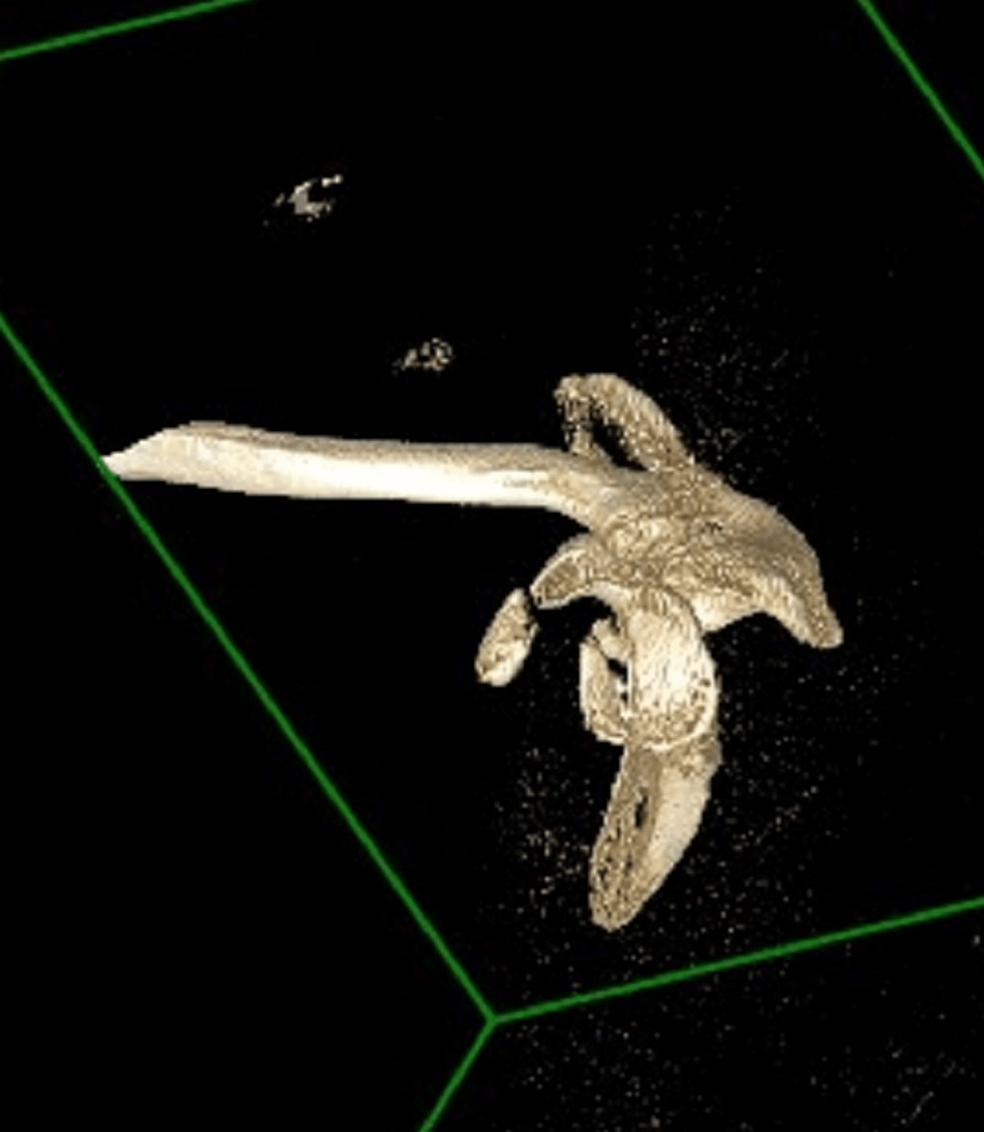
CT reconstruction of the glenoid and coracoid fractures This demonstrates the sizeable anterior glenoid fragment and its comminution, as well as the large coracoid process fragment, which had been displaced.

**Figure 4:**
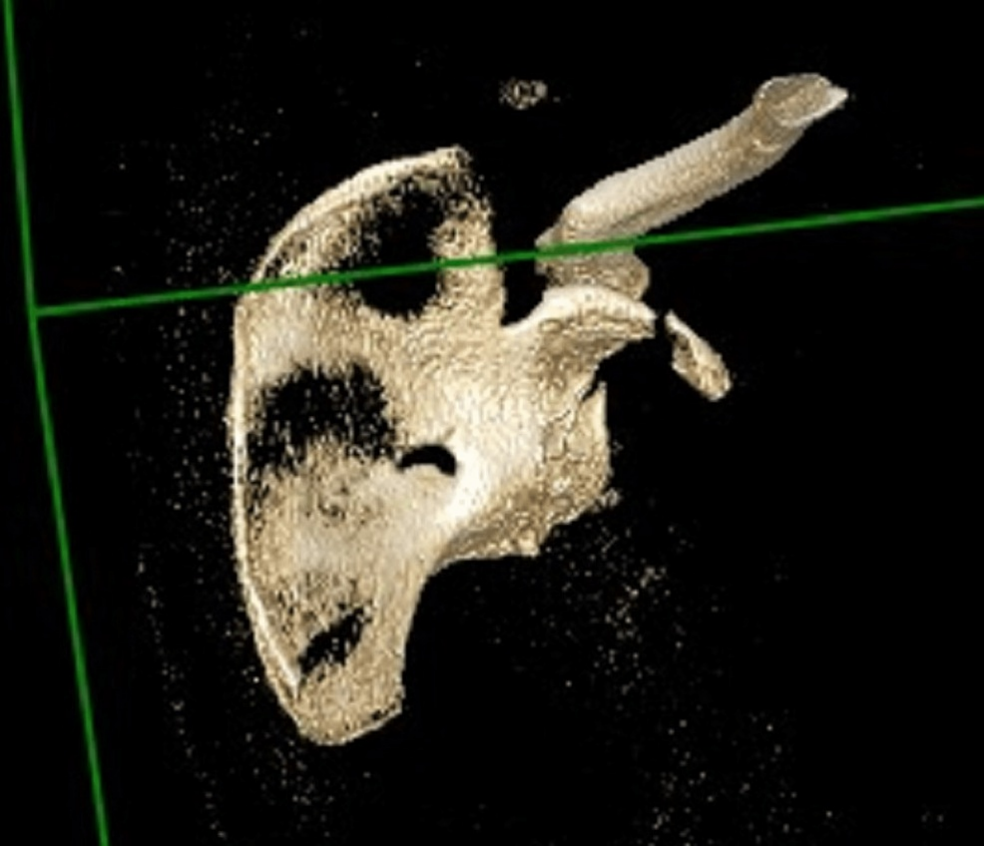
CT reconstruction of the coracoid process fracture Viewed from the anterior, this image demonstrates the large coracoid process fracture and the relevant displacement.

**Figure 5:**
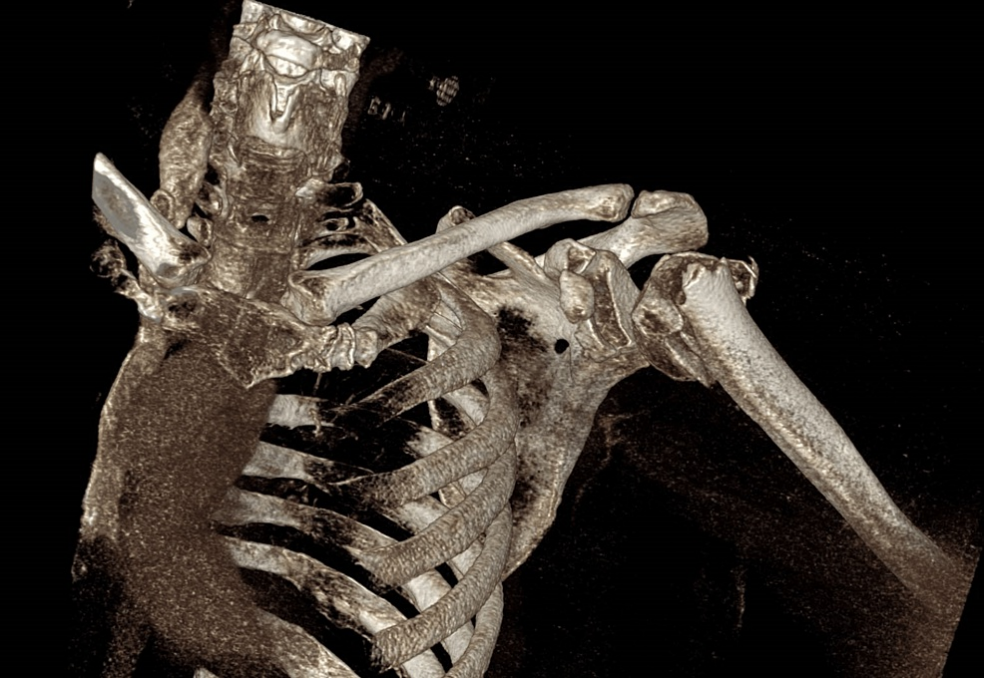
CT reconstruction demonstrating the entire injury pattern without subtraction of other bony structures

He was referred to our on-call trauma service, and due to the high energy involved in his injury and the severe pain that the patient was in, along with the comminuted fracture pattern of the proximal humerus injury, a CT scan was arranged (Figures 2-5).

**Figure 6:**
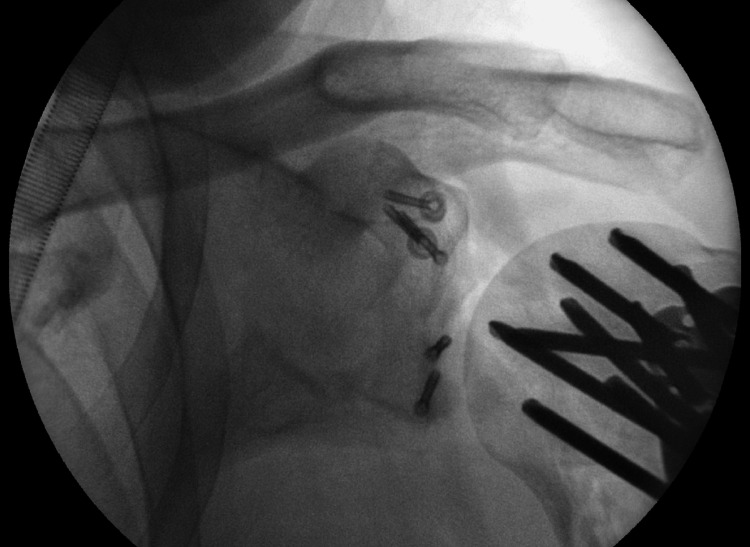
Intraoperative anteroposterior view of the glenohumeral joint This demonstrates the final fixation of the proximal humerus, glenoid, and coracoid processes through a deltopectoral approach.

This confirmed the proximal humerus fracture, but also a significant anterior glenoid fragment that was displaced and comminuted, and a displaced coracoid process fracture. Given the significant displacement of the proximal humerus fracture and the significant instability that this combination of pathologies would confer, we proceeded with the surgical fixation on the next available upper limb specialty trauma list, which was six days following presentation. We used the deltopectoral approach, as we would be able to address all pathologies through this. We started the reconstructive procedure with an open reduction and internal fixation of the proximal humerus fracture, which was comminuted at the metaphyseal level, and the shaft had lacerated the deltoid muscle and had ‘button-holed’ through it. We used size two FiberWire sutures and a long PHILOS locking plate construct. At that point, we performed an examination under anaesthesia and found the glenohumeral joint to be very unstable anteriorly. We subsequently performed a subscapularis split and capsulotomy and stabilised the glenoid anterior wall/rim fragment with two cannulated 2.5mm screws, under direct vision of the joint surface. Our surgical approach at this stage was similar to the one that we use for an open Latarjet procedure. The glenoid fragment was found to have metaphyseal comminution, and we directed our screws just deep into the subchondral plate in order to avoid loss of reduction. After the glenoid fixation, we addressed the coracoid process fracture with anatomical reduction and stabilisation with three 2.5 mm cannulated screws, two of them with washers and one without a washer (Figure 6). The wound was closed in layers, and the patient was placed in a sling in a position of internal rotation.

Postoperative rehabilitation consisted of a sling to be worn for six weeks, with the onset of physiotherapy immediately after surgery, passive range of movement exercises for two weeks, and active-assisted exercises for the four weeks after that. We limited external rotation to the neutral position for six weeks following surgery. Two months following his operation, he had no postoperative complications, but he had a stiff shoulder, a presentation that was expected at that stage after the operation and considering the complex injury pattern.

**Figure 7:**
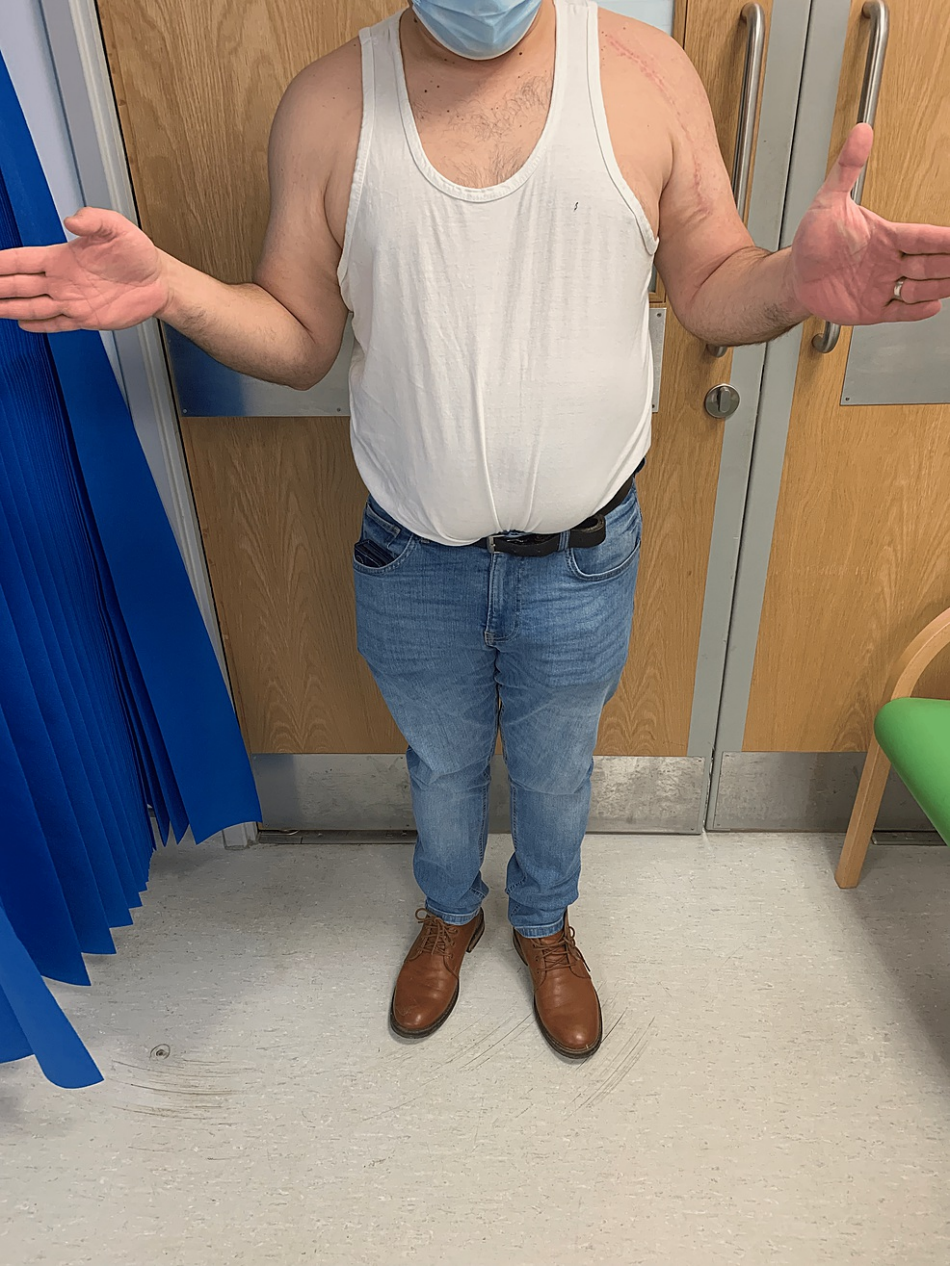
Clinical examination at 14 months after surgery The patient's external rotation was around 30 degrees from neutral.

**Figure 8:**
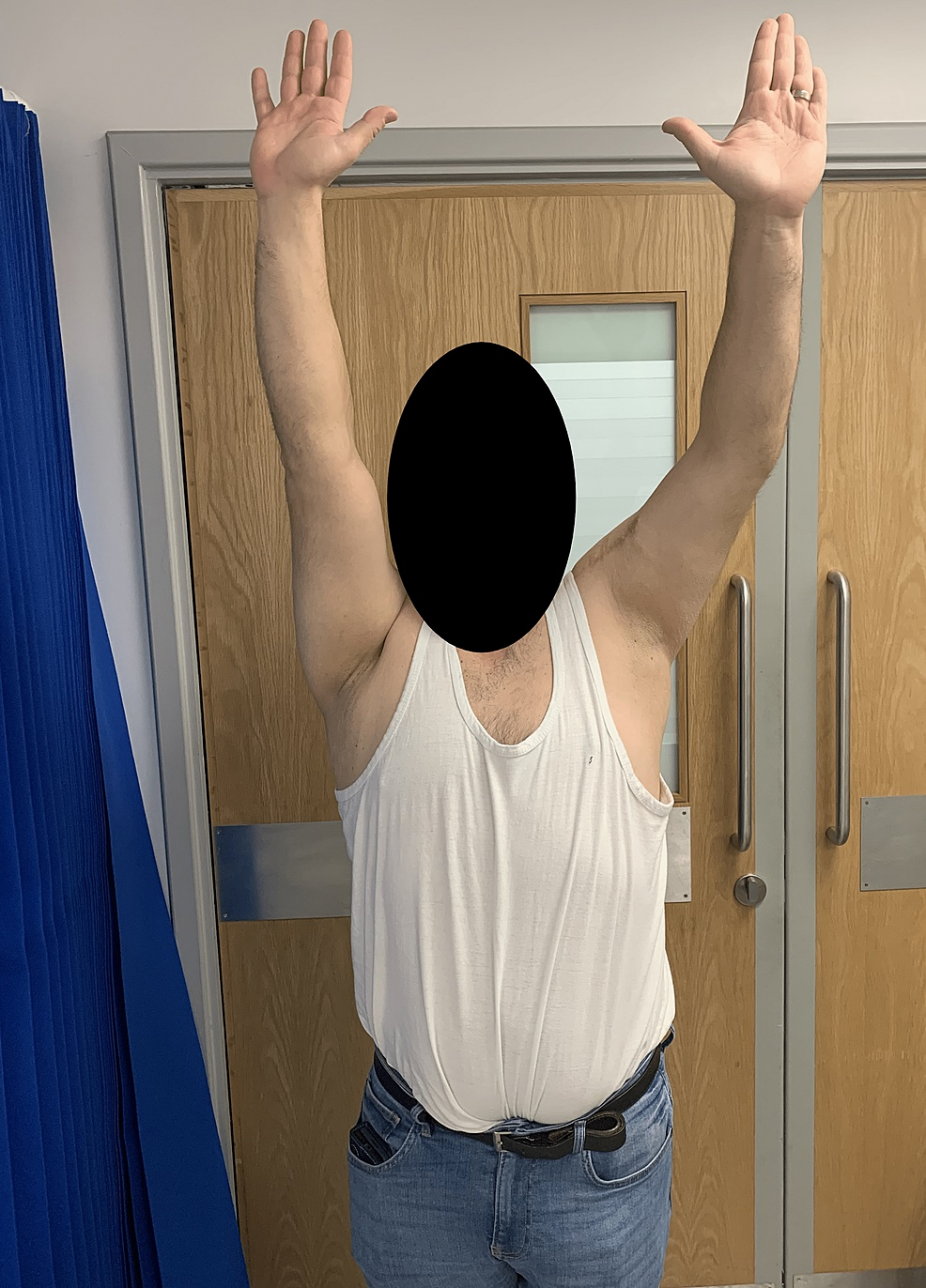
Clinical examination at 14 months after surgery The patient's forward elevation was around 140 degrees on the left side.

**Figure 9:**
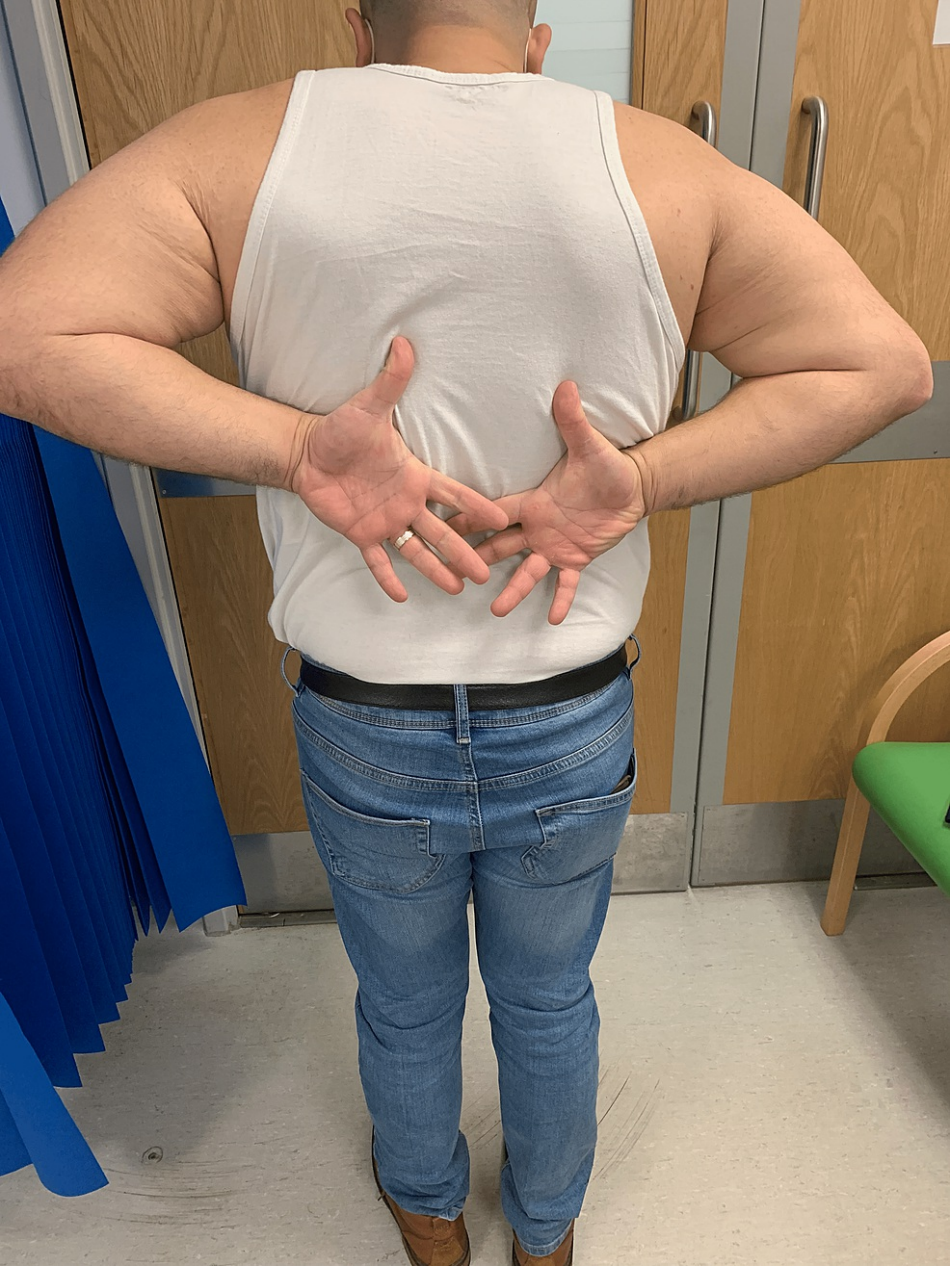
Clinical examination at 14 months after surgery The patient's internal rotation was symmetrical to the contralateral side.

At his four-month follow-up appointment, he had an improving range of movement and no pain, and finally, at 14 months after surgery, he had an external rotation of 30 degrees from neutral, almost normal internal rotation, a forward elevation of 140 degrees (Figures 7-9), no crepitus from the joint, and an Oxford Shoulder Score of 45/48.

**Figure 10:**
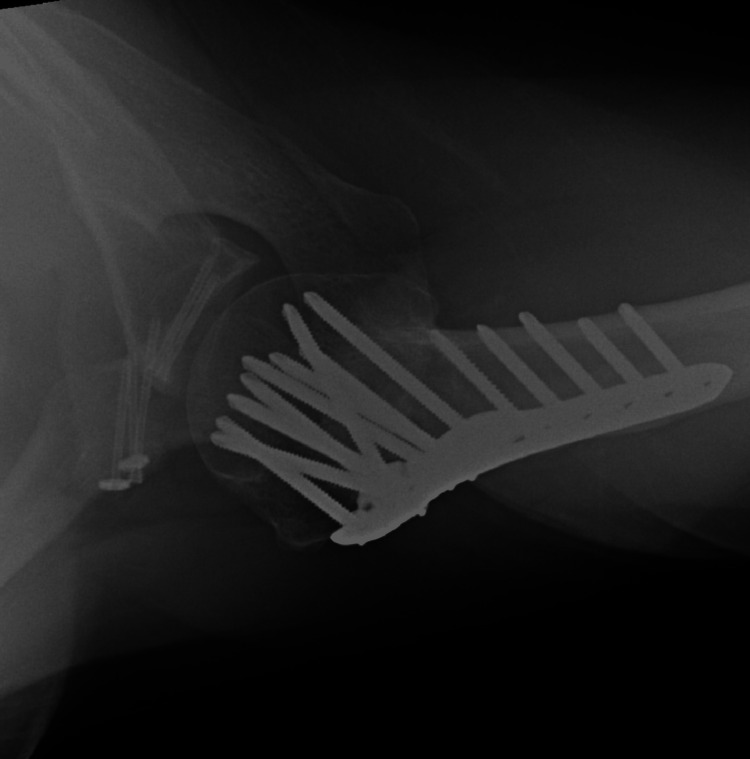
Postoperative axial radiograph at 14 months after surgery The union of all three fractures is evident, and the glenohumeral joint remains concentric.

His shoulder was entirely stable at that point, with a negative anterior apprehension and relocation test. The patient has returned to his previous job. Radiographs taken 14 months after surgery have demonstrated the sound union of all fractures in a satisfactory position (Figure 10).

## Discussion

Proximal humerus fractures in isolation are very common injuries, with a rising incidence of around 100 per 100,000 person-years [3,4]. Several classification systems exist for these injuries, and the Neer classification [5] and AO classification systems are the most commonly used ones. Treatment strategies vary between non-operative and operative management, and these have been heavily researched, with a large level-one randomised controlled trial suggesting that surgical management does not result in a better outcome at two years after surgery when compared to non-surgical management [6].

Rarely, proximal humerus fractures are associated with glenoid fractures. This combination may be indicative of an instability event at the time of injury and is often treated surgically [7], especially when the glenoid fragment is displaced. Although there is no universal consensus for the surgical indications to stabilise these fractures, Königshausen et al. have proposed that surgery is indicated when the glenoid fragment presents with a medial displacement of more than 3 mm and/or a fracture gap of more than 5 mm [7].

Coracoid fractures are also very rare, and when they do get diagnosed, it is often in the presence of a concomitant acromioclavicular joint dislocation [2]. It has been suggested, though, that many coracoid fractures, either isolated or in combination with other pathologies, go undetected as they are not readily visible on antero-posterior shoulder radiographs. They have been classified by Ogawa into four types [8], and their importance was highlighted by the introduction of the concept of the superior shoulder suspensory complex (SSSC) by Goss [9]. Coracoid process fractures introduce a disruption to the SSSC, which, in the presence of additional injuries, may result in unsatisfactory functional outcomes when treated non-surgically [10]. Our patient’s coracoid fracture would be classified as type two using the Ogawa classification system and represents a single disruption in the SSSC.

Glenohumeral combination fractures were described as an entity and classified by Könignhausen et al. [7], who described four possible types. Type 1b describes a large glenoid rim fracture, a greater tuberosity fracture, and an additional coracoid process fracture. There has been a case report [2] and a small case series [11] of type 1b fractures, but our reported case is the first one in the literature that describes fractures to the coracoid, glenoid, and proximal humerus rather than just the greater tuberosity, resulting in a significant disruption to the osseous and soft tissue structures of the shoulder girdle. We would therefore suggest a modification to the Könignhausen classification system to include a type 2b, which our case would fit into.

We approached this case surgically, as detailed above, for a few specific reasons. Stabilising the humerus first restored the anatomy in the shoulder and re-tensioned the subscapularis tendon, such that we were able to perform a subscapularis split in the appropriate location for the subsequent fixation of the glenoid fracture. Dealing with the coracoid fracture last was beneficial because it increased the exposure of the anterior wall of the glenoid, and if the glenoid fragment was found to be more comminuted than expected, we would have been able to utilise the coracoid fragment as a bone block to augment the anterior glenoid. Our patient’s treatment has resulted in a very good functional outcome and range of movement.

## Conclusions

This is a case that serves to reinforce the point that upper limb trauma may yield complex and novel fracture patterns. Treating surgeons need to remain vigilant in such cases, especially in high-energy trauma in young patients. Early and appropriate use of CT scans at the time of presentation to the ED is becoming standard practice and rightly so, as becomes evident from this case. His initial radiographs did not demonstrate the glenoid and coracoid fractures clearly, but an early CT demonstrated this complex injury pattern and resulted in early and appropriate management, which resulted in a satisfactory functional outcome. Based on this case, we propose an additional subtype to the existing classification system for glenohumeral combination fractures.
